# Yeast *XRS2* and human *NBN* gene: Experimental evidence for homology using codon optimized cDNA

**DOI:** 10.1371/journal.pone.0207315

**Published:** 2018-11-15

**Authors:** Ilja Demuth, Simon K. Krebs, Véronique Dutrannoy, Christian Linke, Sylvia Krobitsch, Raymonda Varon, Christine Lang, Andreas Raab, Karl Sperling, Martin Digweed

**Affiliations:** 1 Charité –Universitätsmedizin Berlin, corporate member of Freie Universität Berlin, Humboldt-Universität zu Berlin, and Berlin Institute of Health, Lipid Clinic at the Interdisciplinary Metabolism Center, Berlin, Germany; 2 Berlin-Brandenburg Center for Regenerative Medicine (BCRT), Charité University Medicine Berlin, Berlin, Germany; 3 Institute of Biotechnology, Technical University Berlin, Berlin, Germany; 4 Charité –Universitätsmedizin Berlin, corporate member of Freie Universität Berlin, Humboldt-Universität zu Berlin, and Berlin Institute of Health, Institute of Medical and Human Genetics, Berlin, Germany; 5 Otto Warburg Laboratory, Max Planck Institute for Molecular Genetics, Berlin, Germany; 6 ORGANOBALANCE GmbH, Berlin, Germany; Tulane University Health Sciences Center, UNITED STATES

## Abstract

The genes, *XRS2* in *Saccharomyces cerevisiae* and *NBN* in mammals, have little sequence identity at the amino acid level. Nevertheless, they are both found together with MRE11 and RAD50 in a highly conserved protein complex which functions in the repair of DNA double-strand breaks. Here, we have examined the evolutionary and functional relationship of these two genes by cross-complementation experiments. These experiments necessitated sequence correction for specific codon usage before they could be successfully conducted. We present evidence that despite extreme sequence divergence nibrin can, at least partially, replace Xrs2 in the cellular DNA damage response, and Xrs2 is able to promote nuclear localization of MRE11 in NBS cells. We discuss that the extreme sequence divergence reflects a unique adaptive pressure during evolution related to the specific eukaryotic role for both Xrs2 and nibrin in the subcellular localisation of the DNA repair complex. This, we suggest, is of particular relevance when cells are infected by viruses. The conflict hypothesis of co-evolution of DNA repair genes and DNA viruses may thus explain the very low sequence identity of these two homologous genes.

## Introduction

The efficient repair of DNA double-strand breaks (DSBs) is critical for limiting the mutation rate and preventing genetic imbalance and malignancy. Two-sided DSBs are the most dangerous lesions caused by ionising radiation and one-sided DSBs can also occur during DNA replication. Two major pathways have been identified which are responsible for DSB repair: non homologous end-joining (NHEJ) and homologous recombination repair (HRR). Interestingly, HRR is the major DSB repair pathway in yeast whilst in vertebrates, NHEJ predominates [1]. Components of both pathways have been identified and studied in considerable detail; common to both is the complex of MRE11 and RAD50.

The MRE11/RAD50 complex has both structural and enzymatic functions in DNA repair. The complex has several nucleolytic activities [2] and binds ATP [3]. Scanning force microscopy indicates that the complex forms a bridge structure to tether the ends of double stranded DNA molecules together [4], a prerequisite for both NHEJ and HRR. Furthermore, the complex is also involved in signal transduction by activating the central DNA damage response protein, ATM, leading to cell cycle arrest or apoptosis [5, 6].

MRE11 and RAD50 are highly conserved, in the bacteriophage T4 they are represented by gp47 and gp46 and in *E*. *coli* by SbcD and SbcC [7, 8]. Homologues of MRE11 and RAD50 are also found in the archaea [9], however, an additional, third component of the complex, Xrs2 in yeast and nibrin, coded by the gene *NBN (NBS1)* in mammals, is exclusive to eukaryotes [10]. The association of nibrin with MRE11 and RAD50 is essential for its nuclear localization [11], modifies its enzymatic activity [12] and promotes DSB induced activation of ATM [13]. Surprisingly Xrs2 and nibrin have only 19.3% identity at the amino acid level. In comparison, yeast and human MRE11 sequences are 31.5% identical and RAD50 sequences, 28.8%. This low level of sequence identity between Xrs2 and nibrin has even led to the suggestion that whilst MRE11 and RAD50 are ancient proteins, Xrs2 is an evolutionarily recent development in yeast and entirely lacks homologues in other species [14]. On the other hand, the detection of tandem BRCT domains in both nibrin and Xrs2 suggests that they are indeed true homologues [15].

A hypomorphic mutation of *NBN* in humans is responsible for the chromosomal instability syndrome, Nijmegen Breakage Syndrome [16], in which radiosensitivity, immunodeficiency and early development of haematological malignancy are major features [17]. Null mutation of *Nbn* is lethal in the mouse [18], however, nibrin with the hypomorphic mutation can rescue null mutant cells and mice [19, 20]. In order to examine the functional equivalence of Xrs2 and nibrin despite their weak sequence similarity, we decided to attempt cross-complementation of human and yeast cells. These experiments, however, necessitated sequence correction for specific codon usage before they could be successfully conducted. We then found partial cross-complementation of the DNA damage response in both systems which suggests maintenance of functionality despite recent and rapid sequence divergence. We discuss that this may reflect co-evolution of DNA repair genes and DNA viruses which are known to deregulate the host cell’s DNA repair in order to optimise their own replication [21].

## Materials and methods

### Cells and cell culture

The SV40 immortalized human NBS fibroblast cell line GM7166VA7 [22], homozygous for the *NBN* founder mutation c.657_661del5, GM00637 SV40 immortalized human control fibroblasts and HEK293 cells were cultured in Dulbecco’s Minimal Essential Medium with 10% fetal bovine serum and antibiotics at 37°C, 5% CO_2_.

### *XRS*2 and *NBN* expression constructs

The *XRS2* ORF optimised for expression in mammalian cells using the GeneOptimizer algorithm [23] was synthesized and subcloned in an appropriate vector by Mr Gene (Regensburg, Germany). This construct was used for subcloning into the vector pCMV-Tag2b (Stratagene) allowing the expression of a Flag-tagged version of Xrs2 under control of the CMV promoter. Similarly, the *XRS2* (WT) and *NBN* ORFs were also ligated into pCMV-Tag2b. All constructs were verified by direct sequencing. Enzymes *Sal*I and *Not*I were used to cut the *XRS2* and *NBN* ORFs from the pCMV-Tag2b constructs which were than treated with DNA PolymeraseI and ligated into the *Hpa*I site of pLXIN (Clontech, USA, Mountain View, CA). The DNA sequence of all ORFs was verified by Sanger sequencing.

### Transfection and retroviral transduction

For transfection of plasmids pCMV-Tag2b-*NBN* and pCMV-Tag2b-*XRS2* (wild type and codon optimised), 5×10^5^ HEK293 or GM7166VA7 cells were plated in 2 ml of medium without antibiotics in 6-wells. Cells were transfected the next day with 1.5 μg plasmid DNA using Fugene 6 (Roche) according to the manufacturer’s instructions and analysed 24 h later as described below.

### Immunoprecipitations, immunoblots and immunofluorescence

Immunoprecipitates were prepared by lysing transfected cells in 50 mM Tris–HCl pH 7.5, 150 mM NaCl, 5 mM EDTA and 0.3% Triton X-100 containing a protease inhibitor mixture (Roche Applied Science). Lysates were immunoprecipitated with a Flag-antibody (Sigma-Aldrich) and Dynabeads Protein G (Invitrogen) for 3 h as previously described [24]. Immunoprecipitates were washed four times with lysis buffer and proteins were eluted from the beads by boiling for 5 min.

Lysates and immunoprecipitates were electrophoresed using the NuPage system (Invitrogen) in 4–12% Bis–Tris gradient gels. Following electrophoresis, proteins were transferred to Invitrolon PVDF membranes (Invitrogen). Membranes were blocked for at least 1 h in 10% non-fat milk in Tris-buffered saline, pH 7.6, with 0.1% Tween-20 (TBS-T). Incubation with primary and secondary antibodies was performed in 5% non-fat milk in TBS-T. All washing steps were carried out using TBS-T. Immunoblots were probed with the following primary antibodies: MRE11 (Abcam), RAD50 (Abcam) and Flag-tag, M2 (Sigma-Aldrich). Primary antibodies were detected with horseradish peroxidase-conjugated goat anti-rabbit IgG or goat anti-mouse IgG (BD Pharmingen, San Diego, CA, USA). Chemiluminescence was developed using Western Lightning (PerkinElmer Life Sciences, Boston, MA, USA).

For yeast proteins expressing codon optimised nibrin, yeast cells were collected from suspension by centrifugation washed in phosphate buffered saline and frozen at -80°C. The cell pellets were thawed and lysed by vortexing with glass beads in the presence of PMSF on ice. The lysates were cleared by centrifugation and the supernatant proteins separated on 4–12% polyacrylamide gel (NuPage) as above. Nibrin was detected using the murine monoclonal antibody 1D7 (Abcam) and murine monoclonal anti α-tubulin DM1A (Abcam) as a loading control.

As a functional endpoint, the subcellular localisation of MRE11 was examined. For this cells were fixed, permeabilised and immunostained for MRE11 with a murine polyclonal antibody (Abcam). The primary antibody was detected with goat anti-rabbit IgG coupled to Alexa 568 (Molecular Probes, Eugene, OR) and counter stained with DAPI, as previously described [25]. Immunofluorescence was examined under the Axiophot microscope (Zeiss). Nuclei with fluorescent signals clearly above the background fluorescence in cells transfected with empty vector were considered positive for nuclear MRE11. For quantification a minimum of 300 nuclei were scored in two independent experiments.

### Quantitative RT-PCR

RNA was extracted from HEK293 cells transfected with the pCMV-Tag2b plasmids containing the wildtype or the codon optimised version of the *XRS2* ORF using TRizol (Invitrogen). First-strand cDNA was synthesised from 2 μg RNA using SuperScript III First-Strand Kit (Invitrogen). Real-time PCR was performed with the ABI 7500 PCR System and using POWER SYBR GREEN PCR Master Mix (Applied Biosystems). All samples were analysed in triplicate and copy numbers were calculated by absolute quantification using primers (forward for *XRS2* and *XRS2*syn pCMV-Tag2B-qPCR-F: 5´-GGATGACGACGATAAGAGCC-3´, reverse *XRS2-*qPCR_R: 5´-GAAGGCCTGAAGACAACATGA-3´ and *XRS2*syn-qPCR_R: 5´-GCAGGCAGCAAGAAATGAAGG-3´). For absolute quantification the plasmids were diluted to generate standard curves from 10^9^ copies to 10^2^ copies. Equal loading was controlled by comparing the CT-Values of HPRT (F:5´-AAAACAATGCAGACTTTGCTTTCC-3´; R:5´-AAGTCTGGCTTATATCCAACACTTCG–3´).

### Expression of *XRS2* and *NBN* in yeast and cell survival assays

The ORF of *XRS2* was amplified using genomic DNA isolated from the reference yeast strain BY4741. The amplified DNA fragment was purified and cloned into the cloning vector pJET1.2/blunt. Subsequently, plasmid DNA was treated with the restriction endonucleases *Bam*HI and *Xho*I, the respective DNA fragment purified and subcloned into the *Bam*HI / *Xho*I sites of p416GPD [26] which carries the yeast GPD1 promoter and CYC1 terminator. The sequence of the final construct was validated by sequencing. The *NBN* ORF was optimised for expression in yeast using the GeneOptimizer algorithm, synthesized and subcloned in an appropriate vector by Mr Gene (Regensburg, Germany). The codon optimised sequence was subcloned into p416GPD as above.

For the complementation experiments the *S*. *cerevisiae* XRS*2* deletion strain Y04205 was transformed with the p416GPD constructs by the lithium acetate method [27]. As a control, wild type BY4741 strain Y00000 was transformed with the empty p416GPD vector. For the cell survival assays on plates, yeast cells grown to the stationary phase (72 h) in WMVIII medium [28] supplemented with histidine (100 mg/L), leucine (400 mg/L) and methionine (100 mg/L) were spotted at different dilutions (10^−1^–10^−5^) on YPD plates (10 g/L yeast extract, 20 g/L Bacto Peptone, 20 g/L glucose) that contained either 4 μM camptothecin (CPT) or 15 mM hydroxyurea (HU). Plates were incubated for 24 h at 30°C after spotting. For the UV survival assay the plates were exposed to UV radiation at a dose of 6 mJ (cm^2^)^-1^ after spotting. Subsequently the plates were incubated in the dark for 72 h at 30°C before counting the colony forming units.

## Results

**Fig 1:**
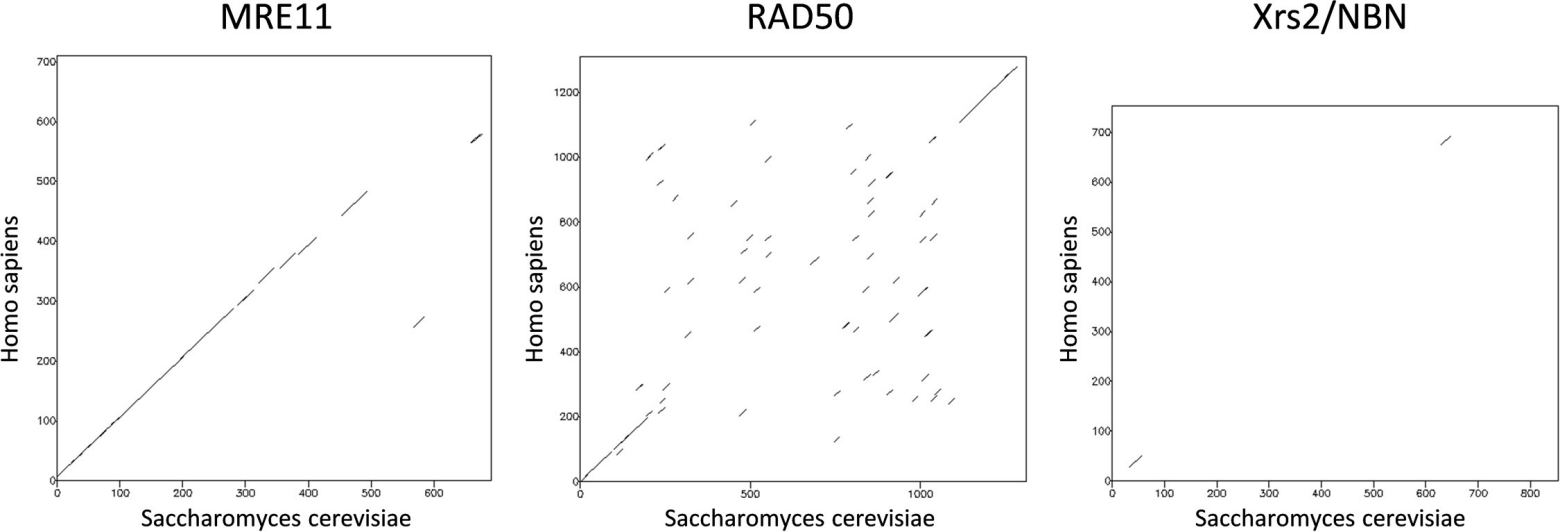
Comparison of the yeast and human MRE11, RAD50 and Xrs2/nibrin amino acid sequences. The figure compares the given sequences by dot plots constructed using the dotmatcher algorithm at EMBOSS (The European Molecular Biology Open Software Suite, [29]). In the plots, a dot indicates that in a sliding window of 15 amino acids the similarity threshold score of 30 was exceeded. Substantial similarity is indicated when the dots align to form diagonal lines.

Whilst the amino acid sequences of MRE11 and RAD50 are highly conserved, the sequences of human nibrin and yeast Xrs2 show only weak similarity. Comparison of the yeast and human sequences using dotmatcher, an algorithm designed to detect regions of sequence similarity [29] shows barely any sequence similarity whilst MRE11 and RAD50 show the conserved regions expected for homologues (Fig 1). Using the Needleman-Wunsch global alignment algorithm [30], identities (and similarities) between yeast and human sequences are 31.5% (46.9%) for MRE11, 28.8% (51.1%) for RAD50 but only 19.3% (34.3%) for Xrs2/nibrin.

**Fig 2:**
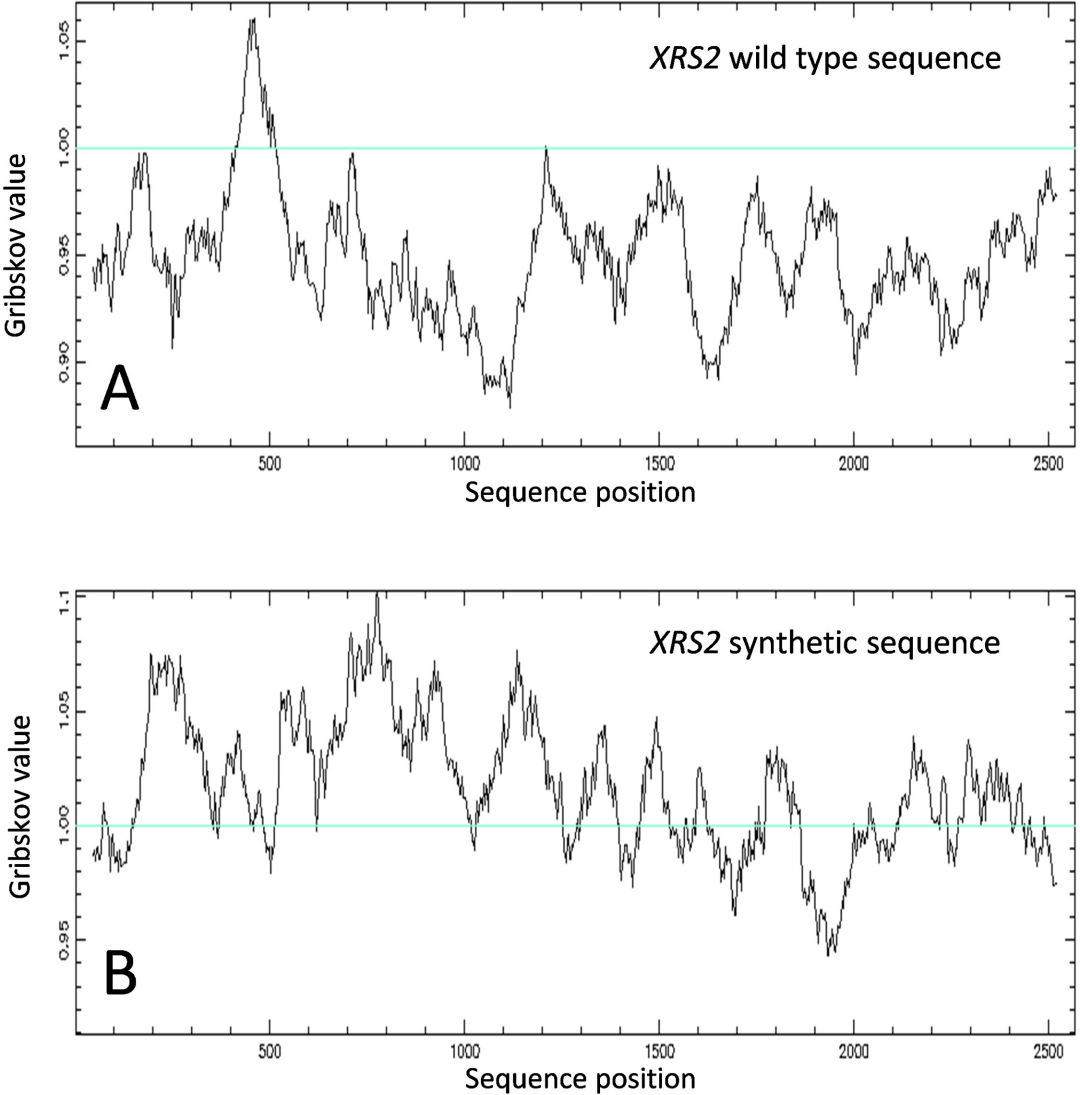
Optimisation of codon usage for expression of yeast *XRS2* in human cells. **A:** The plot shows the Gribskov values for human codon usage calculated for *S*. *cerevisiae* wild type *XRS2* mRNA, the profile is mostly below 1, the value representing normal human codon usage. **B:** Gribskov values for a synthetic *XRS2* sequence designed to maintain amino acid identity but optimise for human codon usage.

**Fig 3:**
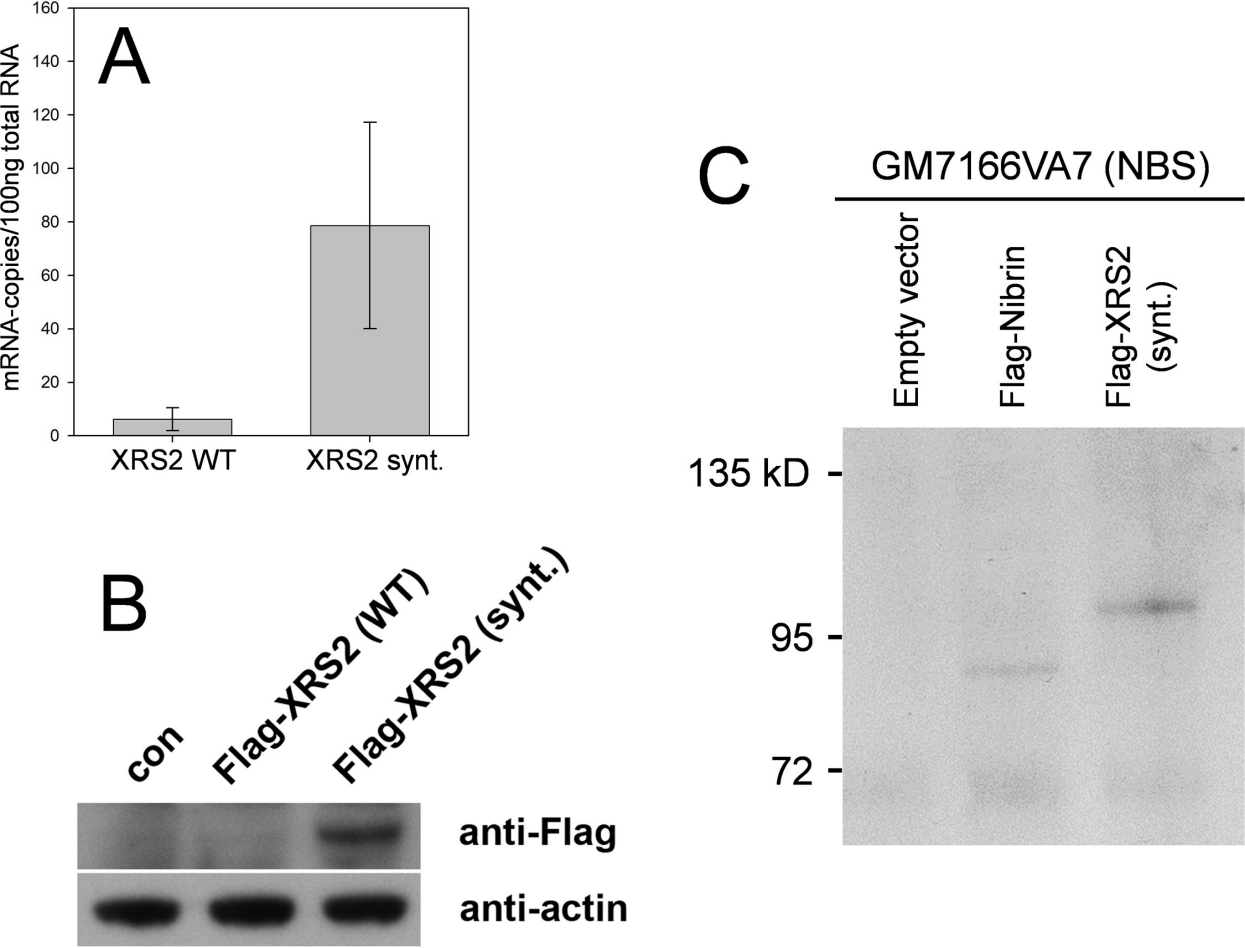
Expression of codon optimized *XRS2* cDNA in human cells. **A:** Realtime quantitative PCR was used to determine mRNA expression in HEK293 cells transiently expressing either Flag-tagged wild type *XRS2* or Flag-tagged synthetic codon-optimised *XRS2*. Expression of the codon optimised mRNA is about 10-fold higher than the wild type mRNA (mean of three independent experiments). **B**: Immunoblot analysis with anti-Flag antibodies to detect Xrs2 protein in HEK293 cells transiently expressing either Flag-tagged wild type *XRS2* or Flag-tagged synthetic codon-optimised *XRS2* cDNA. Actin was detected as a loading control. **C**: Expression of Flag-tagged nibrin and codon optimized synthetic *XRS2* in GM7166VA7 cells, probed by co-immunoprecipitation and western blot analysis using, anti-Flag antibodies.

Our first attempts to express *S*. *cerevisiae XRS2* in human cells, both transiently and stably, were not successful. We reasoned that this might reflect differences in codon usage between human and yeast and indeed, as shown in Fig 2, codon usage in wild type *XRS2* is far from optimal for human cells. We therefore synthesized a codon optimised *XRS2* cDNA for further experiments and, as shown in Fig 3A and 3B this *XRS2* is indeed well expressed in human cells. Interestingly the synthetic sequence leads to a much more stable mRNA, presumably reflecting the rapid degradation of wild type *XRS2* mRNA after discharge from the ribosome due to stalling during translation. Moreover, both Flag-tagged–Xrs2 and Flag-tagged nibrin are efficiently expressed at comparable levels in human NBS cells after transfection (Fig 3C).

**Fig 4:**
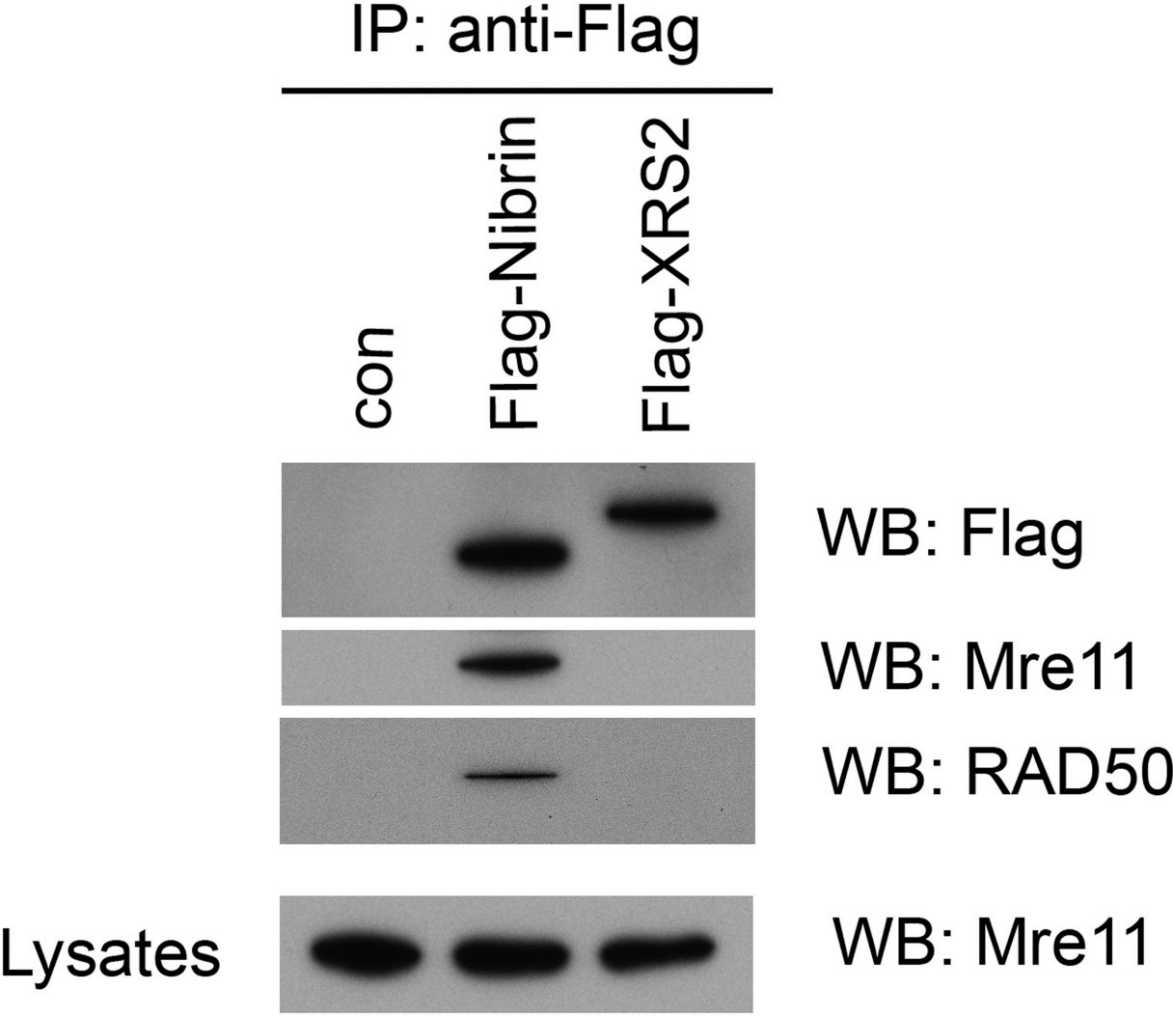
Interaction between MRE11, RAD50 and Xrs2/Nibrin. HEK293 cells transiently expressing Flag-tagged nibrin or codon optimised synthetic X*rs2* were probed by co-immunoprecipitation using anti-Flag antibodies and western blot analysis for RAD50 and MRE11. As a control, aliquots of the lysates were analysed for MRE11 by immunoblotting.

**Fig 5:**
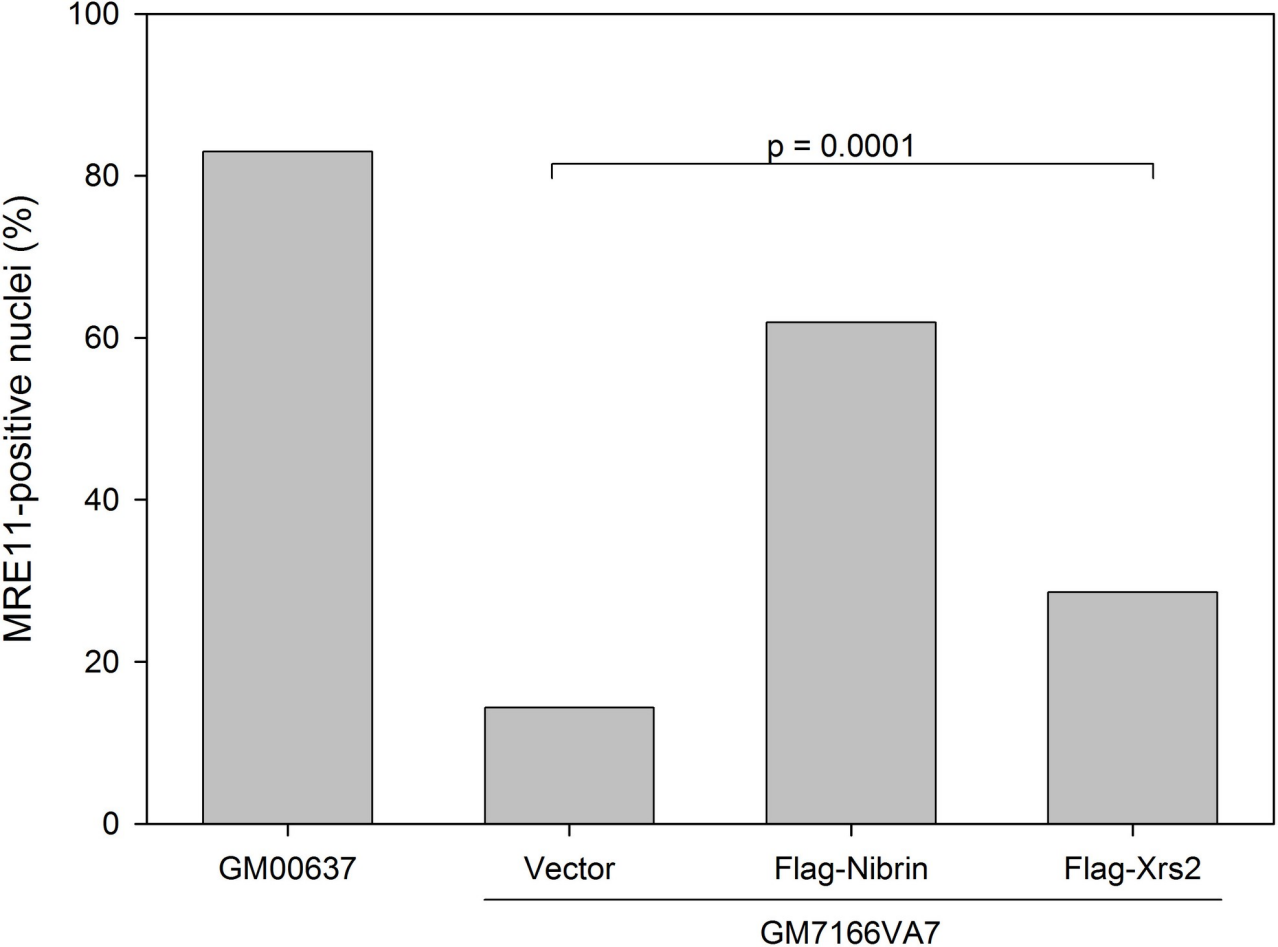
Nuclear localisation of MRE11 in human transfectants. Control SV40 immortalised human fibroblasts (GM00637) and immortalised fibroblasts from a patient with Nijmegen breakage syndrome (GM7166VA7) transiently expressing Flag-tagged full-length nibrin or codon optimised Xrs2 were fixed, permeabilised and stained *in situ* with an antibody directed against human MRE11 followed by a Cy3-labelled secondary antibody. Cells were counterstained with DAPI. Cells transfected with an empty vector were also examined. Quantification of nuclear localisation of MRE11 was performed by scoring a minimum of 300 nuclei for Cy3 nuclear fluorescence in two independent experiments. Results from each of the two experiments were tested for significance using the Fisher’s exact test (two-tailed) and were shown to be significant with p-values below 0.01. The graph displays pooled data from the two experiments.

The functionality of yeast *XRS2* in human cells was further addressed by examining its binding to the other components of the complex, human MRE11 and RAD50. As shown in Fig 4, transiently expressed nibrin co-immunoprecipitated RAD50 and MRE11 whilst codon optimised synthetic *XRS2* did not. Since co-immunoprecipitation may require particularly strong and durable protein association, we also examined the interaction of Xrs2 with MRE11 using a cellular assay. MRE11 is normally entirely nuclear but in cells from NBS patients it is mostly cytoplasmic [11]. Similarly, in the C-terminal domain of Xrs2 there is a MRE11 binding site required for translocation of MRE11 to the nucleus in yeast [31]. As shown in Fig 5, expression of both nibrin and codon optimised Xrs2 leads to a shift in MRE11 localisation to the nucleus, although the effect after ectopic expression of Xrs2 was lower than after nibrin. This indicates that the interaction between Xrs2 and MRE11 is weaker or rarer than the interaction between nibrin and MRE11. Indeed, as shown in Fig 4, we were unable to co-immunoprecitipate MRE11 or RAD50 along with Flag-tagged Xrs2 from human cells.

**Fig 6:**
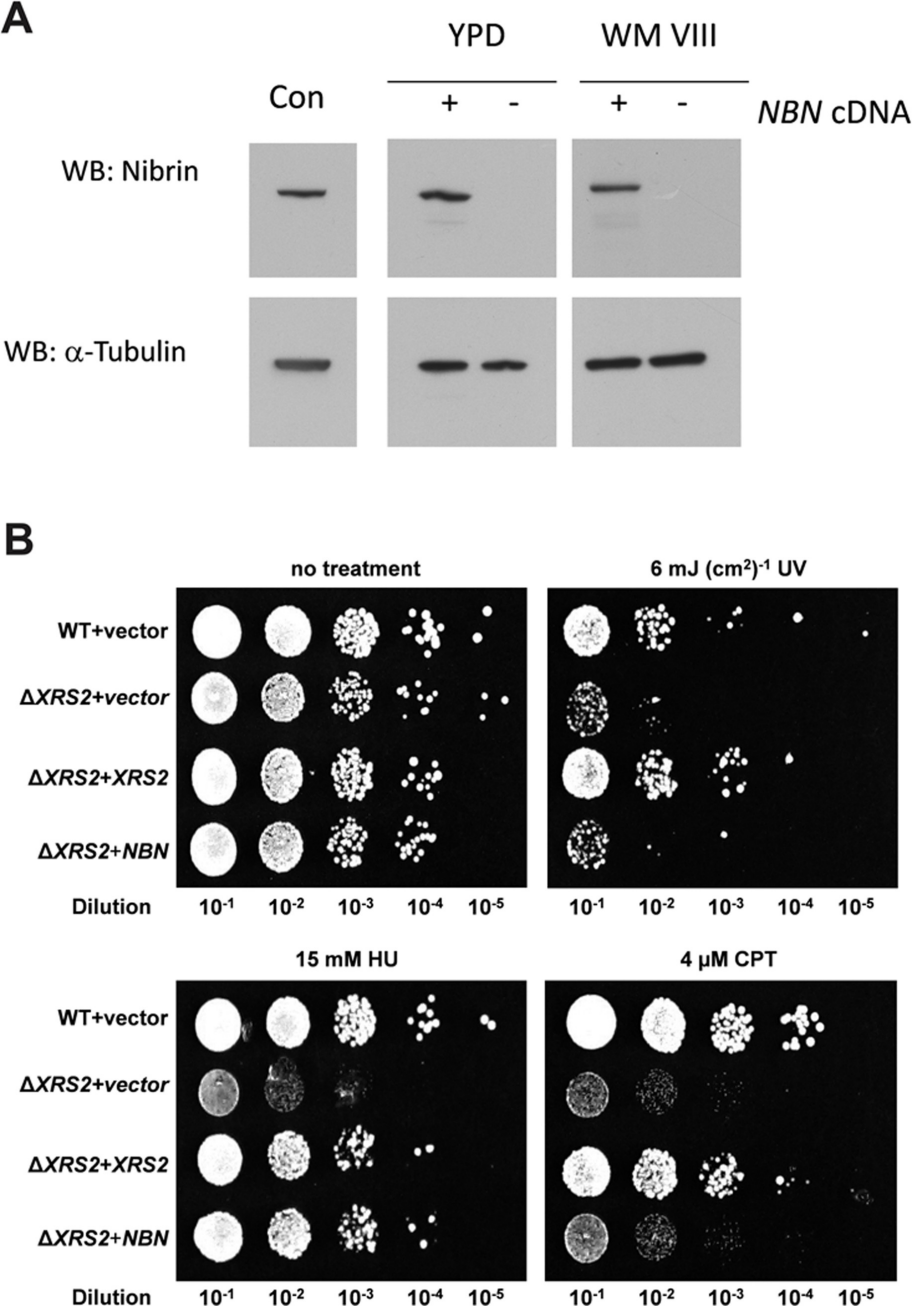
Yeast sensitivity to UV, hydroxyurea and camptothecin in the absence of XRS2 and correction by human nibrin expression. **A:** Y04205 yeast cells, in which the *XRS2* gene is deleted, were transfected with plasmid p416GPD carrying human nibrin cDNA codon optimised for expression in yeast (+) or with empty p416GPD vector (-). Cells were grown for 15 hours in YPD full medium or for 72 hours in selective WMVIII medium, lysed and protein lysates examined by western blot for nibrin expression. ‘Con’ is a cell lysate from human fibroblasts. The blot was reprobed with an antibody directed against α-tubulin as a loading control. **B:** Yeast strains Y00000 (WT) and Y04205 (Δ*XRS2*) transformed with the empty p416GPD plasmid and Y04205 transformed with p416GPD carrying the yeast *XRS2* gene (XRS2) or human *NBN* cDNA codon optimised for expression in yeast (*NBN*) were grown for 72 hours in selective WMVIII medium. Cultures were spotted on YPD plates in the absence or presence of hydroxyurea (HU) or camptothecin (CPT). For evaluation of sensitivity to UV the plates were exposed to 6 mJ (cm^2^)^-1^ after spotting. Each row represents a dilution series (10^−1^–10^−5^, left to right). Plates were incubated for 48 h after spotting the dilution.

Fig 6A shows the expression of codon optimised *NBN* in yeast Y04205 cells, a mutant in which the *XRS2* gene is deleted. Since there is robust expression of nibrin in these cells we went on to examine correction of the DNA repair defect. The functionality of *NBN* in mutant Y04205 yeast cells was examined in dilution survival experiments. Y04205 cells are sensitive to single stranded lesions after UV radiation and to DNA DSBs caused by hydroxyurea and topisomerase I inhibitors such as camptothecin. As shown in Fig 6B, UV sensitivity of Y04205 yeast cells is hardly affected by expression of human nibrin. In contrast, sensitivity to hydroxyurea is strongly reduced by NBN expression and sensitivity to camptothecin is weakly corrected (compare 10^−3^ and 10^−4^ dilutions between *XRS2* mutant and *NBN* expressing yeast cells in Fig 6B). The difference in response to the two mutagens, which has been noted previously [32], reflects the different mechanisms of DSB generation. Hydroxyurea depletes cellular dNTPs and leads to replication fork stalling and subsequently DSBs. This is in contrast to induction of DSBs by irradiation or radiomimetic drugs such as camptothecin which inhibits Topoisomerase 1. This more direct mechanism invokes the cellular DNA damage response, including phosphorylation of nibrin by ATM, unlike DSB induction by hydroxyurea [32]. Phosphorylation of nibrin by ATM related Tel1 in yeast may be incomplete. It has previously been shown that mutations in *XRS2* have different consequences for hydroxyurea and camptothecin sensitivity [33], illustrating the importance of protein sequence for these repair pathways.

## Discussion

Using multiple sequence alignment of 25 sequences from *xrs2* to *NBN* allowed Becker et al. to detect tandem BRCT domains in Xrs2 and nibrin despite less than 10% sequence identity in the 250 amino acid sequence examined [15]. The structural and functional significance of the second BRCT domain in nibrin could be demonstrated by nuclear magnetic resonance spectroscopy and *in situ* mutagenesis studies [34] suggesting that *NBN* and *XRS2* are true homologues with functional equivalence.

We present here evidence that yeast Xrs2 and human nibrin show significant functional cross-complementation. However, the considerable sequence divergence suggests that evolution has led to adaptation of these particular components of the MR(X-N) complex. This may reflect differences in the relative use of NHEJ and HRR in yeast and mammals. DSB repair by HRR is considered to be the more ancient pathway [14] so that the divergence of nibrin could reflect the preference of mammalian cells for NHEJ which can be ascribed to several differences between yeast and higher eukaryotes. Firstly, the increased size, complexity and packing of the genome will hamper the search for homologous sequences during HRR. Secondly, in continually dividing unicellular yeast cells, a sister chromatid is available for HRR for most of the time. In comparison, differentiated mammalian cells are no longer in the cell cycle and thus have no identical sequence for HRR. Finally, the development of an immune system where DSBs are a prerequisite for V(D)J recombination and immune class switching. It has been directly shown, also in a conditional *Nbs* null mutant mouse model, that the MRN complex is involved in immune class switching which is based on the NHEJ pathway [35–38]. Clearly, these rearrangements necessitated adaptation of the processing pathway for DSBs to avoid HRR.

Adaptation of DSB processing is apparently particular to *XRS2* and *NBN* rather than *MRE11* or *RAD50*. Rapid adaptive evolution specifically of *XRS2* and *NBN* has been previously reported. Using a bioinformatic screen in *Saccharomyces* species to search for genomic regions with an excess of non-synonomous nucleotide changes over synonymous changes, identified 72 genes which have evolved under positive selection. Two biological processes were especially enriched for such genes: meiosis and NHEJ [39]. In particular, between *S*. *cerevisiae* and *S*. *paradoxus*, *XRS2* showed 12 non-synonomous changes to one synonymous change whilst *MRE11* and *RAD50* showed no non-synonomous changes. Extending the analysis to seven *Saccharomyces* species yielded strong, statistically significant support for positive selection over neutral evolution in *XRS2* but not in *MRE11* or *RAD50* [40]. In a similar analysis of primate NHEJ genes, five genes were found to have been under recent adaptive evolution one of which is *NBN* [41]. As in the *Saccharomyces* study, positive selection of *MRE11* and *RAD50* was not observed under primate NHEJ genes.

Why is it that *XRS2* and *NBN* should be under such strong selective pressure? We and others have previously reported that the MRN response to DNA damage is altered in cells infected with the SV40 virus due to expression of the large T antigen [25, 41] and it has been demonstrated that SV40 infection leads to proteasomal degradation of the MRN complex [42]. Indeed, it has become clear that viral manipulation of the cellular DNA repair response is a general feature of viral DNA replication and a promoter of tumorigenesis (reviewed in [21]). Thus it has been suggested that an evolutionary conflict between viruses and NHEJ genes has led to rapid evolution of key components of the DNA repair pathway [40]. A similar argument has been made for retrotransposons and evolution of NHEJ genes in yeast [39]. In cells infected with adenovirus 5 with a deletion in the E4 gene, promotion of DSB joining by the MRN complex leads to concatenation of linear viral DNA and inhibition of its packaging into virus particles [43]. In cells infected with wild type adenovirus 5, the MRN complex is found in cytoplasmic aggregates as a preliminary step in its proteasomal degradation [43, 44]. Similarly, the LANA protein of Kaposi Sarcoma Herpesvirus recruits the MRN complex and modulates viral latency [45].

Nibrin is of particular importance for the correct nuclear localisation of the MRN complex for DNA repair [11, 46] and likely also for viral replication. For example, early in infection, ATM and the MRN complex localise to the nuclear sites of HSV-1 replication [47]. Similarly, the MRN complex localizes to viral replication compartments as a result of lytic reactivation of Kaposi's Sarcoma-Associated Herpesvirus [48]. In consequence, sequence changes in *NBN* which reduce interaction with viral proteins whilst maintaining cellular DNA repair function have likely been beneficial and thus rapidly selected. Experimental evidence for this has been given by the report of Lou et al. [49] who compared the binding of gibbon and siamang nibrin to the HSV-1 protein, ICP0. Just four amino acid differences between these two nibrin sequences had major effects on both ICP0 binding and virus production. The amino acid changes lead to a loss of ordered structure in nibrin, reduced interaction with ICP0 and prevented requisition by the HSV-1 virus [49]. Successful interaction of viral proteins with nibrin might be particularly important for viruses such as the *Poxviridae*, which replicate in the cytoplasm [50, 51], In conclusion, nibrin’s function in localisation of the MRN complex may be the clue to its rapid sequence divergence.
